# Status of pesticide management in the practice of vector control: a global survey in countries at risk of malaria or other major vector-borne diseases

**DOI:** 10.1186/1475-2875-10-125

**Published:** 2011-05-14

**Authors:** Henk van den Berg, Jeffrey Hii, Agnes Soares, Abraham Mnzava, Birkinesh Ameneshewa, Aditya P Dash, Mikhail Ejov, Soo Hian Tan, Graham Matthews, Rajpal S Yadav, Morteza Zaim

**Affiliations:** 1 Laboratory of Entomology, Wageningen University, P.O. Box 8031, 6700EH Wageningen, the Netherlands; 2 World Health Organization, Regional Office for the Western Pacific, Manila, Philippines; 3 World Health Organization, Regional Office for the Americas, Washington DC, USA; 4 World Health Organization, Regional Office for the Eastern Mediterranean, Cairo, Egypt; 5 World Health Organization, Regional Office for Africa, Harare, Zimbabwe; 6 World Health Organization, Regional Office for South-East Asia, New Delhi, India; 7 World Health Organization, Regional Office for Europe, Copenhagen, Denmark; 8 21 Lorong Abang Openg Lima, Taman Tun Dr Ismail, 60000 Kuala Lumpur, Malaysia; 9 Imperial College, Ascot, UK; 10 Vector Ecology and Management, Department of Control of Neglected Tropical Diseases, World Health Organization, Geneva, Switzerland

## Abstract

**Background:**

It is critical that vector control pesticides are used for their acceptable purpose without causing adverse effects on health and the environment. This paper provides a global overview of the current status of pesticides management in the practice of vector control.

**Methods:**

A questionnaire was distributed to WHO member states and completed either by the director of the vector-borne disease control programme or by the national manager for vector control. In all, 113 countries responded to the questionnaire (80% response rate), representing 94% of the total population of the countries targeted.

**Results:**

Major gaps were evident in countries in pesticide procurement practices, training on vector control decision making, certification and quality control of pesticide application, monitoring of worker safety, public awareness programmes, and safe disposal of pesticide-related waste. Nevertheless, basic conditions of policy and coordination have been established in many countries through which the management of vector control pesticides could potentially be improved. Most countries responded that they have adopted relevant recommendations by the WHO.

**Conclusions:**

Given the deficiencies identified in this first global survey on public health pesticide management and the recent rise in pesticide use for malaria control, the effectiveness and safety of pesticide use are being compromised. This highlights the urgent need for countries to strengthen their capacity on pesticide management and evidence-based decision making within the context of an integrated vector management approach.

## Background

Malaria and other vector-borne diseases continue to inflict a major burden on human populations [1-3]. The control of malaria has been intensified in the last decade, relying to a substantial degree on the action of chemical pesticides to control vector populations or reduce disease transmission. It is critical that these vector control pesticides are used for their acceptable purpose without causing adverse effects on health and the environment. The International Code of Conduct on the distribution and use of pesticides, hereafter referred to as the Code of Conduct, provides voluntary standards for all public and private entities engaged in, or associated with, the distribution and use of pesticides, and serves as a globally-accepted standard for pesticide management [4].

Countries at risk of malaria and/or other vector-borne diseases, however, face major challenges in managing vector control pesticides and other public health pesticides, which include pesticides for use by households and pest control operators [5]. The challenge is greatest under decentralized health systems. Many countries, even those with long-standing vector control programmes [6], lack capacity to regulate availability and use of the pesticides for vector control purposes. Proper management of pesticides throughout their life cycle, from product development to waste disposal, requires legislation, regulatory control, operational guidelines and procedures, criteria, training, safety measures, quality control, informing the public, and evaluation [7].

The state of implementation of the Code of Conduct with a focus on agricultural pesticides has been reported in 1993, 1996 and 2008 [8-10]. The results suggest that in the past 15 years there has been progress in some areas, such as data collection, labelling, storage and the establishment of poison control facilities. However, little progress was reported in pesticide quality control, removal of hazardous products from the market, integrated pest management, resistance management, monitoring of adverse effects, and safe disposal of pesticide-related waste.

Comparable studies on management of public health pesticides have been lacking. A preliminary study on public health pesticides conducted in 2003 indicated various shortcomings in countries, but the scope of the questionnaire was rather limited and the coverage modest, with 71 responding countries [11]. Based on the experience of the preliminary study, an improved and a more comprehensive study was conducted with the aim to provide an overview of the global status on management of public health pesticides. The purpose was to inform future plans on the optimization and harmonization of public health pesticide registration procedures and post-registration regulation among countries, and to assist countries in developing strategies and action plans for strengthening capacity on pesticide management.

**Figure 1:**
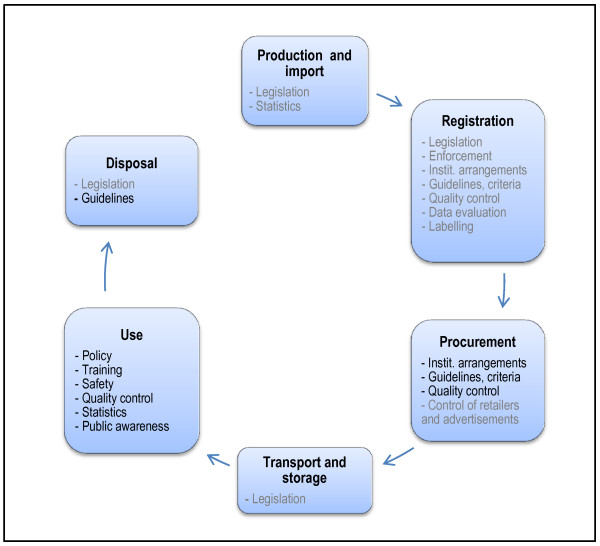
**Life cycle of public health pesticides**: The stages, from product development to waste disposal, with aspects of pesticide management pertaining to each stage. Black text indicates management aspects addressed in this paper; grey text indicates aspects addressed in a related paper [12]

This paper reports on part of the questionnaire, which relates to the practice in vector control (Figure 1); a separate paper reports on legislation and regulatory control [12]. In-depth assessment of specific aspects or quantitative comparison between Regions or countries was beyond the scope of this assessment.

## Methods

The questionnaire had been developed through a consultative process of the World Health Organization (WHO), with field testing in selected countries and peer review [13]. Countries targeted were those member states of the WHO, territories excluded, which are endemic with or at risk of malaria or one of the other major vector-borne diseases, i.e. lymphatic filariasis, dengue, leishmaniasis, Chagas disease, and Japanese encephalitis. Hence, the study excluded most of the European countries (except for 6 countries), North America, Japan, Australia and New Zealand.

The questionnaire was distributed through six WHO Regional Offices to 142 countries [13]. The questionnaire was translated into three languages; the English version was administered to most countries except where the French or Spanish version was preferred. Focal points for malaria and other vector-borne diseases in WHO representative's offices facilitated the data collection in each country through the ministry of health. The part of the questionnaire addressed in this paper was completed by, either, the director of the vector-borne disease control programme, or the national manager for vector control. Most questions relate to vector control pesticides but a few questions refer more generally to public health pesticides (Table 1).

**Table 1:** The questionnaire

	Question		Positive response	n
1	Is there a national vector control unit or core group with the responsibility for all vector control activities?		69%	110
2	Is there a national integrated vector management (IVM) policy for vector-bone disease control?		62%	110
3	Does the Ministry of Health (MoH) use or make reference to the International Code* in the management of public health pesticides?		72%	106
4	Is susceptibility of vectors used as a basis for selection of pesticides?		85%	103
5	Is there a national guidance document for procurement of pesticides for vector control?		52%	110
6	Is procurement of pesticides by MoH, for vector control programmes, centralized?		88%	105
7	Is there any other agency or authority that procures pesticides for vector control?		64%	110
8	Is procurement of vector control pesticide products by the MoH restricted to those recommended by WHOPES?		81%	109
9	Is procurement of vector control pesticide products by the MoH carried out through public tenders?		76%	109
10	Are after-sale stewardship commitments**, incorporated as a condition in procurement of vector control pesticide products?		53%	109
11	Are WHO quality standards for public health pesticide products (i.e. WHO specification) included in procurement requirements by the MoH?		90%	107
12	Is quality control (pre- and/or post-shipment) of vector control pesticide products required for procurements by the MoH?		77%	106
13	Is the use of appropriate personal protective equipment mandatory for vector control pesticide applicators?		87%	108
14	Is personal protective equipment made available to applicators of vector control pesticide operations by the MoH?		87%	107
15	Is there a certification scheme for pesticide applicators in vector-borne disease control programmes?		40%	107
16	Are there any national guidelines for health monitoring of pesticide applicators in vector control operations?		43%	108
17	Is there a national programme to monitor applicator exposure to pesticides used in vector control operations?		26%	107
18	Is there a national scheme for quality control of pesticide application equipment for vector control (including space spray equipment)?		35%	110
19	Are WHO quality standards for vector control pesticide application equipment used in the quality control of such equipment?		67%	109
20	Are records of vector control pesticide product usage available to the MoH at the national (central) level?		79%	110
21	Have those responsible for decision-making and implementation of vector control activities received certified training in:			
	a. vector control?	All	21%	109
		Part	75%	109
		None	4%	109
	b. sound management of public health pesticides?	All	16%	109
		Part	79%	109
		None	5%	109
22	Is there any national information and awareness programme, for the public, on use of public health pesticides?		41%	107
23	Does the MoH have guidance document(s) for disposal of vector control pesticide waste?		42%	107
24	Does the MoH have guidance document(s) for disposal of vector control pesticide containers?		38%	107

Questions as formulated in the questionnaire, with the percentage of countries giving a positive response to each question, and the number of countries responding to each question (n).

*The International Code of Conduct on the Distribution and Use of Pesticides

**E.g. information packages and training

The questionnaire consisted mostly of logical choices between two options (yes/no), but for a few questions there were more options. Responses were entered into a computer spread-sheet. Some open questions were used to further clarify or verify the practices reported but the responses were not included in the analysis. In several cases where the respondent selected more than one option or failed to respond, that record was excluded from analysis.

In the analysis, all targeted countries were given equal weight, irrespective of country size or disease burden, because the focus of the study was on pesticide management at the national level. Country responses were examined per Region.

## Results

In total, 113 countries responded to the questionnaire, showing an 80% response rate (113/142). In terms of human population, these countries represented 94% of the population in all countries targeted, based on demographic data for 2008 [14]. Consequently, the 29 non-responding countries had relatively small populations. Response rates per Region were 65% for the African (30 countries out of 46 targeted), 85% for the American (28/33), 81% for the Eastern Mediterranean (17/21), 83% for the European (5/6), 73% for the South-East Asian (8/11), and 100% for the Western Pacific Region (25/25). The relatively low response rate from the African Region was mainly attributable to logistic issues in some countries.

The aggregated results of the questionnaire show a high variability in the positive responses to each question (Table 1), with 103 to 110 countries responding to individual questions. The aggregated results were used to highlight areas of concern and to suggest steps which could be taken by countries to improve pesticide management. The areas of concern were grouped under seven themes subjected to closer examination in the following sections.

### Policy and coordination

According to country responses, the Code of Conduct is being used, or made reference to, in the management of public health pesticides by the ministry of health in 72% of countries, but in the Eastern Mediterranean, South-East Asian and Western Pacific Regions this was around 60% of countries (Table 2).

**Table 2:** Status of policy and coordination in the WHO Regions

	Use of the Code		IVM policy		Vector control unit		Usage records	
	
WHO Region	%	n	%	n	%	n	%	n
African	83%	29	53%	30	60%	30	73%	30
American	76%	25	78%	27	89%	27	93%	27
Eastern Mediterranean	56%	16	71%	17	56%	16	76%	17
European	80%	5	60%	5	60%	5	100%	5
South-East Asian	63%	8	57%	7	88%	8	88%	8
Western Pacific	65%	23	50%	24	63%	24	65%	23
All	72%	106	62%	110	69%	110	79%	110

as expressed by: the use of, or reference made to, the International Code of Conduct in the management of public health pesticides; the presence of a national IVM policy; the presence of a national vector control unit or core group responsible for all vector control activities; and the availability of records on usage of vector control pesticide products to the ministry of health at central level. Presented is the percentage of countries answering each question positively, and the number of responding countries (n), for each Region.

Sixty-two per cent of responding countries reported having a national policy on Integrated Vector Management (IVM) for vector-borne disease control; a national policy was least common in the African and Western Pacific Regions. IVM is a rational decision-making process for the optimal use of resources for vector control [15]. The aim of IVM is to improve the efficacy, cost-effectiveness, ecological soundness and sustainability of vector control [16].

The presence of a national vector control unit or core group with responsibility for all vector control activities was reported from 69% of countries. Such a unit is vital for coordination in planning, implementation, monitoring and evaluation of vector control operations for malaria and other vector-borne diseases. In the African, Eastern Mediterranean, European and Western Pacific Regions, only around 60% of countries reported having such unit.

On average, 79% of countries reported having records on vector control pesticide product usage available to the ministry of health at central level (Table 2). Information on usage -which ideally includes pesticide type, formulation, dosage, amount, locations, as well as records on the effectiveness of applications-is crucial to providing guidance and support to vector control operations and pesticide resistance management. Lack of usage records was most prominent in the African, Eastern Mediterranean and Western Pacific Regions.

These four aspects of policy and coordination could be considered important underpinnings of a national system of vector control, which were reported present in the majority of countries. Thirty-five percent of countries answered positively to all four questions and another 33% to three out of four questions (n = 102).

### Procurement

In most countries (88%), pesticide procurement for vector control programmes by the ministry of health is centralized, which can be considered favourable for the efficient use of resources. Nevertheless, 64% of countries reported that other agencies or authorities were also involved in pesticide procurement (Table 1).

Procurement is a highly specialized area, and national guidelines and procedures on procurement are essential for ensuring transparency, fostering healthy competition and facilitating access to good quality pesticide products. Just over half of all countries reported having such guidance document, and in the Western Pacific Region where many countries are small-island states this was available in only 38% of countries (Table 3).

**Table 3:** Status of procurement procedures for vector control pesticide products in the WHO Regions

	Guidance document		Public tenders		After-sale stewardship	
	
WHO Region	%	n	%	n	%	n
African	50%	30	86%	29	48%	29
American	46%	26	70%	27	50%	26
Eastern Mediterranean	59%	17	81%	16	53%	17
European	80%	5	100%	5	80%	5
South-East Asian	88%	8	100%	8	63%	8
Western Pacific	38%	24	54%	24	54%	24
All	52%	110	76%	109	53%	109

as expressed by: the presence of a national guidance document for procurement; procurement by the ministry being carried out through public tenders; and after-sale stewardship commitments as a condition in procurement. Presented is the percentage of countries answering each question positively, and the number of responding countries (n), for each Region.

Procurement through public tenders has the advantage that government can stipulate conditions for stewardship support, while obtaining pesticides at competitive prices and in a transparent manner. Public tenders were reported by 76% of countries but were least common in the Western Pacific Region (Table 3).

Only 53% of countries responded that after-sales stewardship commitments such as provision for information packages and training were incorporated as a condition in the tenders for procurement of vector control pesticide products (Table 3). Manufacturers are best positioned, and indeed urged under the Code of Conduct, to advise governments on the lifecycle management of their products.

Quality control of vector control products, pre- and/or post-shipment, which is critical to the effectiveness, efficiency and safety of interventions, was reported being a procurement requirement in 77% of countries (Table 1). Quality control should be conducted by independent and accredited laboratories, and should include the determination of active ingredient content and relevant physical and chemical pesticide properties as per specifications such as those developed by WHO, where available.

### Training

Planning, implementation, monitoring and evaluation of vector control require specific knowledge and skills. In only a minority of countries (21%) have all of those responsible for decision making and implementation of vector control received certified training on vector control or on sound management of public health pesticides (Table 4). In 75-79% of countries only part of the persons at responsible positions had received training on these relevant topics while in 4% of countries none had received this training.

**Table 4:** Status of training in the WHO Regions

	Training on vector control				Training on pesticide management			
	
WHO Region	All	Part	None	n	All	Part	None	n
African	7%	86%	7%	29	7%	86%	7%	30
American	41%	59%	0%	27	23%	77%	0%	26
Eastern Mediterranean	35%	59%	6%	17	29%	53%	18%	17
European	0%	100%	0%	5	25%	75%	0%	4
South-East Asian	13%	74%	13%	8	13%	87%	0%	8
Western Pacific	13%	82%	5%	23	8%	88%	4%	24
All	21%	75%	4%	109	16%	79%	5%	109

as expressed by: certified training in vector control and in sound management of public health pesticides received by those responsible for decision making and implementation of vector control activities. Presented is the percentage of countries specifying whether all, part, or none have received training, and the number of responding countries (n), in each Region.

### Pesticide application

Two aspects of pesticide application need highlighting. First, the effectiveness and safety of major vector control interventions depends on the application technique, requiring specific skills. However, a certification scheme for pesticide applicators in vector-borne disease control programmes was reported from only 40% of all countries and in just 25% of countries in the American and Eastern Mediterranean Regions (Table 5).

**Table 5:** Status of quality control of vector control pesticide application in the WHO Regions

	Certification scheme for applicators		Quality control scheme for equipment	
	
WHO Region	%	n	%	n
African	47%	30	27%	30
American	25%	24	33%	27
Eastern Mediterranean	25%	16	41%	17
European	60%	5	40%	5
South-East Asian	38%	8	75%	8
Western Pacific	54%	24	30%	23
All	40%	107	35%	110

as expressed by: the presence of a certification scheme for pesticide applicators in vector-borne disease control programmes; and the presence of a national scheme for quality control of pesticide application equipment for vector control. Presented is the percentage of countries answering each question positively, and the number of responding countries (n), in each Region.

Second, routine maintenance of equipment is essential to ensure the efficiency, effectiveness and safety of spraying operations. Yet, only a minority of countries (35%) responded having a national scheme for quality control of pesticide application equipment for vector control; the figures are lowest in the African, American and Western Pacific Regions (Table 5). This indicates another gap. In the South-East Asian Region, consisting mostly of large countries with long-standing vector control programmes, 75% of countries responded having such a scheme in place.

Most countries (85%) responded that the susceptibility of vectors was used as basis for selection of pesticides (Table 1). Nevertheless, further details on the comprehensiveness and frequency of susceptibility testing in countries will be needed to substantiate this response. Independent reports suggest that the capacity for pesticides resistance monitoring needs strengthening in many countries [17,18].

### Safety

The use of appropriate personal protective equipment was reportedly mandatory for vector control pesticide applicators in 87% of countries (Table 1). Also, personal protective equipment was reportedly made available to applicators of vector control pesticide operations by the ministry of health in 87% of countries.

Despite these positive figures, availability of national guidelines for health monitoring of pesticide applicators in vector control operations were reported by only 43% of countries, and in the African and Western Pacific Regions this was 33% and 35%, respectively (Table 6). Moreover, only 26% of countries reported having a national programme to monitor applicator exposure to pesticides used in vector control operations. Again, the figures were lowest in the African and Western Pacific Regions.

**Table 6:** Status of safety aspects of public health pesticide management in the WHO Regions

	Health monitoring guidelines		Health monitoring programme		Public awareness programme	
	
WHO Region	%	n	%	n	%	n
African	33%	30	13%	30	40%	30
American	56%	25	44%	25	22%	23
Eastern Mediterranean	41%	17	31%	16	41%	17
European	60%	5	40%	5	60%	5
South-East Asian	50%	8	38%	8	63%	8
Western Pacific	35%	23	13%	23	50%	24
All	43%	108	26%	107	41%	107

as expressed by: the presence of national guidelines for health monitoring of pesticide applicators in vector control operations; the presence of a national programme to monitor applicator exposure in vector control; and the presence of any national information and awareness programme, for the public, on use of public health pesticides. Presented is the percentage of countries answering each question positively, and the number of responding countries (n), in each Region.

Just 41% of countries reported having any national information and awareness programme in place, for the public, on the use of public health pesticides (Table 6). These programmes were least common in the American Region.

### Disposal

Just 42% and 38% of countries responded that their ministry of health has guidance documents for disposal of vector control pesticide waste and vector control pesticide containers, respectively. Guidelines should point out, for example, that used pesticide containers are not used for storing food or drinking water, thus preventing contamination.

### Use of WHO recommendations

Three questions in the questionnaire referred to the use of WHO recommendations (Table 7). The majority of countries (81%) responded that the procurement of vector control pesticide products by the ministry of health is restricted to those recommended by the WHO. Also, 90% of countries responded that WHO-specifications are included in procurement requirements of the ministry of health. Moreover, 67% of countries responded that WHO-quality standards for vector control pesticide application equipment were used in the quality control of such equipment.

**Table 7:** Role of recommendations by the WHO Pesticide Evaluation Scheme in the WHO Regions

	Procurement of re-commended products		Procurement using quality standards		Quality standards for equipment	
	
WHO Region	%	n	%	n	%	n
African	86%	29	96%	28	53%	30
American	81%	27	96%	26	69%	26
Eastern Mediterranean	81%	16	81%	16	76%	17
European	100%	5	100%	5	100%	5
South-East Asian	75%	8	100%	8	75%	8
Western Pacific	71%	24	75%	24	65%	23
All	81%	109	90%	107	67%	109

as expressed by: the restriction of procurement to those vector control products recommended by WHO; the inclusion of WHO quality standards in procurement requirements by the ministry of health; and the use of WHO standards for quality control of equipment for vector control pesticide application. Presented is the percentage of countries answering each question positively, and the number of responding countries (n), in each Region.

## Discussion

These results show the global situation on the management of pesticides in the practice of vector control, serving as baseline for future initiatives to strengthen the management of vector control pesticides. The survey is exceptional in its high coverage of populations at risk of malaria and other major vector-borne diseases.

The study had several limitations. It was assumed that respondents represented institutional memory, but their actual period in office could have limited the accuracy of their responses. The questionnaire did not allow for qualified statements and in-depth interpretation of results. Moreover, the choice to count all countries as equal, rather than weighting country responses according to population size or disease burden, may have introduced a bias; e.g. if large countries have better developed pesticide management systems than small countries. This problem was partly circumvented by examining the country responses per Region; e.g. with the South-East Asian Region consisting of few but large countries, or with the African Region consisting mostly of highly endemic countries.

It is encouraging to observe that several basic conditions of policy and coordination for pesticide management have been established in many countries, which are: the use of the Code of Conduct, a national IVM policy, a national vector control unit, and statistics on pesticide usage. These conditions are indicative of a country's strategy or prospect for improving pesticide management in the practice of vector control.

Nevertheless, the results have exposed some major gaps in pesticide management globally, in particular in the areas of procurement, training, pesticide application, safety and disposal. Also, the authors have noticed that there are different perceptions of the IVM concept in the Regions.

Regarding pesticide procurement for vector control programmes, national guidelines are absent in half of the countries, after-sales stewardship commitment of the manufacturer not required in almost half of the countries, and quality control not required by some countries. Positive aspects are that procurement is mostly through the ministry of health and is often conducted through public tenders. These are conditions through which procurement procedures could potentially be improved, for example by incorporating stewardship commitments on training of applicators on low-risk and appropriate use and safe disposal of waste.

Training on decision making and implementation of vector control is another area of concern, which will be most acute in situations where decision making has been decentralized to the district level. In some countries, none of those responsible had received any training on vector control or public health pesticide management. In its handbook on IVM, the WHO proposes ways to improve the efficacy, cost-effectiveness, ecological soundness and sustainability of vector control [16]. Means to achieve this are an increasing emphasis on local evidence, adoption of a multi-disease approach, and combining vector control interventions wherever appropriate. Processes through which this could be achieved are the integration within the health sector, collaboration between sectors and participation of communities. Clearly, training investment is required, including on pesticide management, to generate skills of analysis, decision making and facilitation at the national, district and village level.

All chemical pesticides are inherently toxic to humans, and precaution is required to minimize exposure and adverse health effects. Applicators and handlers of pesticides may have a particularly high exposure risk if not protected, especially in tropical climates where use of protective equipment is often lacking owing to personal inconvenience. Further study is needed to determine the coverage, quality, use and maintenance of personal protective equipment in relation to the degree of hazard of pesticides used.

Health monitoring of vector control pesticide applicators is not being given due attention in most countries, highlighting another gap. Under the Code of Conduct, governments are obliged to carry out health surveillance programmes of occupationally exposed workers, and to investigate and document poisoning cases. Furthermore, certification schemes for pesticide applicators and national scheme for quality control of equipment for vector control pesticide application were missing in the majority of countries.

Public awareness programmes, lacking in the majority of countries, are needed wherever pesticides are applied in or around houses to promote people's compliance with the interventions. Furthermore, safe disposal of pesticide-related waste was inadequately addressed in the majority of countries; while governments and industry are obliged under the Code of Conduct to cooperate on this topic with the industry to provide stewardship support.

Since 2002, the WHO, through its Pesticide Evaluation Scheme, has expanded its support to member states in the low-risk and judicious use of public health pesticides and their sound management. The survey results show that WHO's recommendations and standards were adopted by most countries, indicating an important advisory and technical support role of the Organization in the management of vector control pesticides globally [12].

The gaps identified in this study highlight the need for further action. Foremost, awareness-raising is needed for policy makers and programme managers about the urgency of good pesticide management and evidence-based decision making within malaria vector control programmes. The WHO has begun to assist a number of countries in conducting an analysis on the national situation on public health pesticide management, and in preparing national action plans to address shortcomings [19,20]. Nevertheless, countries need to mobilize resources and build capacity to implement those plans. International agencies should assist countries in developing policies and adopting international guidelines, facilitate regional collaboration (e.g. on insecticide resistance monitoring) and offer support for capacity building on pesticide management as component of IVM.

## Conclusions

Given the gaps in pesticide management identified in this study, the effectiveness and safety of vector control pesticide use are clearly being undermined in many countries. This inevitably results in wastage of resources, sub-optimal effectiveness of interventions and adverse effects on human health and the environment. The urgency of the situation is emphasized by the recent rise in pesticide use for malaria vector control in countries that have scaled up interventions [1]; a development that has not necessarily been accompanied by investment in pesticide management. Therefore, capacity building on pesticide management and evidence-based decision making within the context of an IVM approach should be incorporated in any malaria vector control programme and should become a condition for support on vector control given by donors and funding agencies.

## Competing interests

The authors declare that they have no competing interests.

## Authors' contributions

HvdB analysed and interpreted the data and drafted and revised the manuscript. JH, AS, AM, BA, APD and ME participated in the design and data acquisition and contributed to revising the manuscript. SHT contributed to the analysis and interpretation of data. GM contributed to revising the manuscript. RSY and MZ conceptualized the study, contributed to the analysis and interpretation of data and to revising of the manuscript. All authors have read and approved the final manuscript.
